# Melatonin influences methyl jasmonate-induced protection of photosynthetic activity in wheat plants against heat stress by regulating ethylene-synthesis genes and antioxidant metabolism

**DOI:** 10.1038/s41598-023-34682-y

**Published:** 2023-05-08

**Authors:** Zebus Sehar, Mehar Fatma, Sheen Khan, Iqbal R. Mir, Gholamreza Abdi, Nafees A. Khan

**Affiliations:** 1 Plant Physiology and Biochemistry Laboratory, Department of Botany, Aligarh Muslim University, Aligarh, 202002 India; 2 Department of Biotechnology, Persian Gulf Research Institute, Persian Gulf University, Bushehr, 75169 Iran

**Keywords:** Plant sciences, Photosynthesis, Plant development, Plant hormones, Plant physiology, Plant stress responses

## Abstract

Melatonin (MT) and methyl jasmonate (MeJA) play important roles in the adaptation of plants to different stress factors by modulating stress tolerance mechanisms. The present study reports the involvement of MT (100 µM) in MeJA (10 µM)-induced photosynthetic performance and heat stress acclimation through regulation of the antioxidant metabolism and ethylene production in wheat (*Triticum aestivum* L.) plants. Plants exposed to 40 °C for 6 h per day for 15 days and allowed to retrieve at 28 °C showed enhanced oxidative stress and antioxidant metabolism, increased 1-aminocyclopropane-1-carboxylic acid (ACC) synthase (ACS) activity and ethylene production, and decreased photosynthetic performance. In contrast, the exogenously applied MT and MeJA reduced oxidative stress through improved S-assimilation (+ 73.6% S content), antioxidant defense system (+ 70.9% SOD, + 115.8% APX and + 104.2% GR, and + 49.5% GSH), optimized ethylene level to 58.4% resulting in improved photosynthesis by 75%. The use of *p*-chlorophenyl alanine, a MT biosynthesis inhibitor along with MeJA in the presence of heat stress reduced the photosynthetic performance, ATP-S activity and GSH content, substantiated the requirement of MT in the MeJA-induced photosynthetic response of plants under heat stress. These findings suggest that MeJA evoked the plant’s ability to withstand heat stress by regulating the S-assimilation, antioxidant defense system, and ethylene production, and improving photosynthetic performance was dependent on MT.

## Introduction

The increasing heat stress issue is an imminent threat to crop productivity all over the world. Heat stress is a condition where the temperature exceeds a certain threshold for an extended period of time resulting in detrimental repercussions on the overall growth and development of plants ^1^. Nevertheless, the threshold value of temperature varies between cell compartments and across the species ^2^. The exploitation of the full genetic potential of plants in a stressful environment has become a challenging task ^3^. The modification in phenological and physiological responses due to heat stress can be used as indicators for monitoring and sensing the severity of heat stress. The consequences of heat stress in plants include disruptions to cellular physiological processes and modifications in molecular mechanisms ^4^. It induces oxidative stress through enhanced formation of reactive oxygen species (ROS) that leads to elevated lipid peroxidation of membranes, protein denaturation and nucleic acid degradation ^4^. In order to overcome heat stress impacts, plants tend to upregulate various defense mechanisms, such as reduction in H_2_O_2_ and TBARS production through upregulation of activity of enzymatic and non-enzymatic antioxidant system and regulation of ethylene synthesis.

Plant hormones/growth regulators exert a potential influence in enhancing plant growth and productivity under optimal and heat stress conditions ^5^. Melatonin (MT, *N*-acetyl-5-methoxy tryptamine), a pleiotropic signaling molecule has been recognized as a potential growth regulator which functions in defense against different environmental constraints ^6^. Likewise, the role of methyl-jasmonate (MeJA) as a potential growth regulator in controlling plant’s response to abiotic challenges, and growth and development has been recognized in many plants ^7,8^. The available literature has shown that exogenous supplementation of MT in wheat seedlings reduced the deleterious effects of high-temperature stress by modifying the antioxidant system ^9^. Similarly, MeJA protected the photosystem system (PS) II in heat-stressed wheat plants by stabilizing the chloroplast D1 protein and increasing the rate of enzymatic antioxidants ^10^. MeJA promoted heat tolerance in perennial ryegrass by altering chlorophyll (Chl) production and degradation, the antioxidant enzyme system, the HSF-HSP network, and jasmonic acid (JA) production ^11^. It has been shown that exogenous MeJA promoted the accumulation of JA and MeJA content under heat stress by elevation of the transcript levels of genes involved in the JA biosynthesis pathway ^11,12^. MT also protected tomato seedlings from heat-induced damage by regulating polyamine and nitric oxide production and restoring redox homeostasis ^13^. Furthermore, MeJA increased the activity of antioxidant enzymes, ascorbate peroxidase (APX), superoxide dismutase (SOD), catalase (CAT) and peroxidase, and reduced the Cd stress-induced oxidative damage ^14^. It has been recorded that MeJA mitigated the arsenic (As)-mediated oxidative stress by regulating the redox state of ascorbate (AsA) and GSH in cultivars of mustard ^15^. Plants treated with MT reduced the effects of cadmium (Cd) stress by promoting the synthesis of thiols (cysteine; Cys, glutathione; GSH, and phytochelatins; PCs), suggesting a potential function for MT in the acquisition of sulfur (S) ^16^. The study of Siddique et al. ^17^ established that MT and S had a synergistic role in mitigating the negative effect of lanthanum toxicity by upregulating the enzymatic antioxidant and mitigating ROS-induced oxidative damage. The studies suggest that there is a strong connection between MeJA and S-metabolism, and through signaling crosstalk with S-assimilation, MeJA can control a variety of plant responses under stressful and optimal situations ^11,11^.

Wheat (*Triticum aestivum* L.), a plant that is commonly cultivated for cereal, occupies second place in terms of the most important stable food for the human population ^18^. *T. aestivum* is highly sensitive to climate factors, which significantly reduces its productivity by heat stress in arid, semiarid, tropical, and subtropical regions of the world ^18^. It responds to heat stress well at temperatures between 17 and 23 °C ^19^ and is impacted by higher temperatures above 30 °C. Heat stress reduced seed germination and impaired wheat plant growth ^20^, which in turn harms photosynthetic apparatus by slowing down electron transport, deactivating the photosystem II center, deteriorating proteins, and ultimately lowering yield. Wheat quality and production are being challenged by recent rises in global average temperature and the frequency of high temperatures. As its’ quality and yield have a direct impact on the food security, new research is needed for their rescue. Ironically, there are few reports available on the impact of MT and MeJA in heat stress tolerance in wheat plants, but no information is available on the influence of MT with MeJA in the promotion of photosynthesis under heat stress in wheat plants through the regulation of ethylene production, and antioxidant metabolism. The mechanisms by which MeJA and MT protect the photosynthetic pigment system, specifically through the regulation of ethylene synthesis-related gene expression in heat-stressed wheat leaves, have not been examined. Therefore, the present study aimed to assess the impact of exogenous MT and MeJA on physio-biochemical responses of wheat plants under heat stress, and to study the involvement of MT in MeJA-induced photosynthetic performance and heat stress acclimation.

## Results

### Impact of MT or/and MeJA on oxidative stress under heat stress

**Figure 1:**
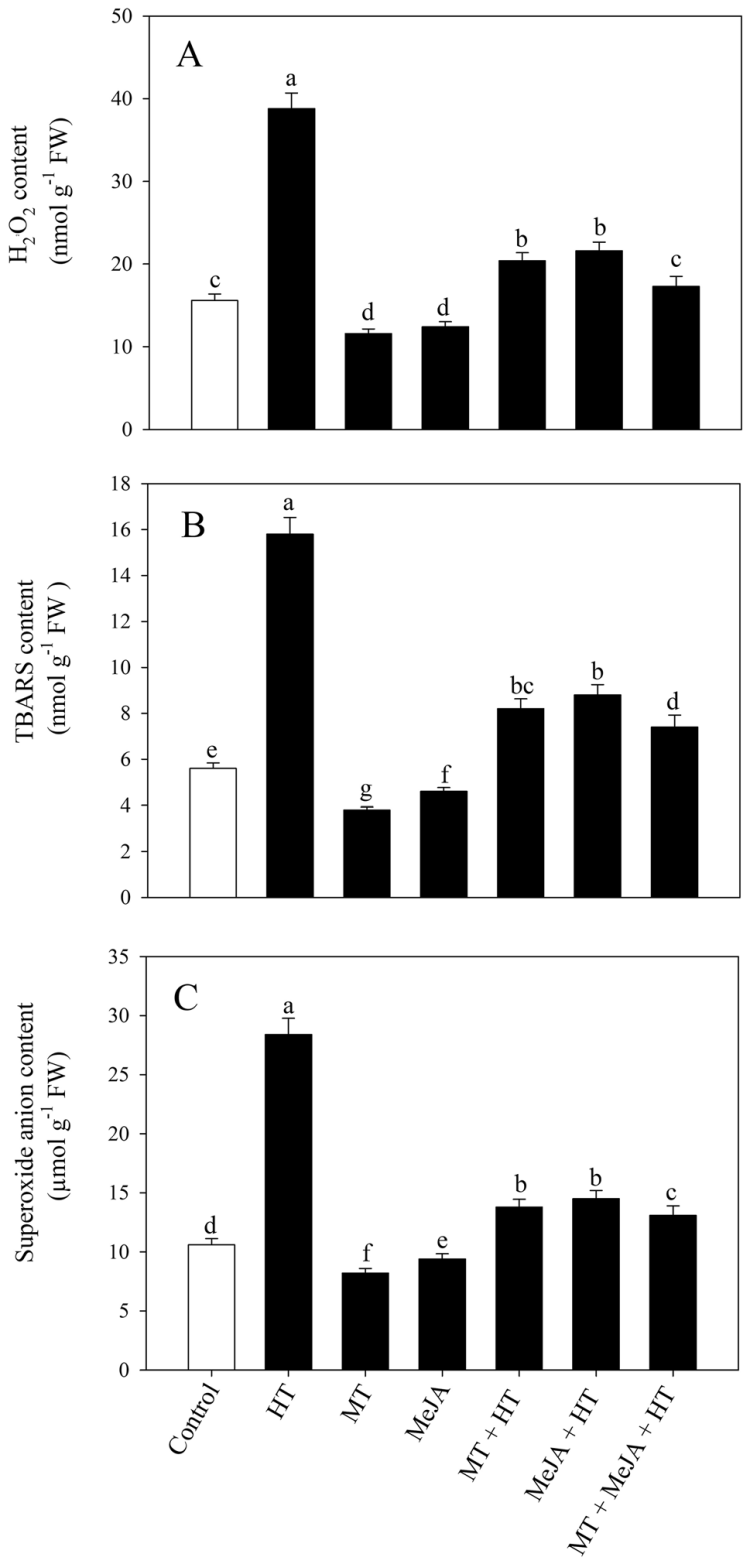
Content of H_2_O_2_ (**A**) and TBARS (**B**) and O^2.−^ (**C**) in wheat (*Triticum aestivum* L.) leaves at 30 DAS. Plants were grown with or without high temperature stress (40 °C for 6 h per day for 15 days), and the foliage was treated with 100 µM MT and/or 10 µM MeJA at 20 DAS. Data are presented as treatment mean ± SE (n = 4). Data followed by the same letter are not significantly different from the LSD test at *p* < 0.05. DAS, days after sowing; HT, heat stress; MT, melatonin; MeJA, methyl jasmonate.

The level of oxidative stress and lipid peroxidation was determined by examining H_2_O_2_, superoxide ion (O^2.−^), and TBARS content (Fig. 1A-C). Compared to control, a sharp increase of 148.7%, 167.9%, and 182.1% was observed in H_2_O_2_, O^2.−^ and TBARS content, respectively, on the exposure to heat stress. In contrast, individual application of MT and MeJA under stressed and unstressed conditions reduced the oxidative stress biomarkers content in plant leaves. Under heat stress, MT supplementation reduced H_2_O_2_ by 47.2%, O^2.−^ by 52.8%, and TBARS by 48.1% relative to only heat-stressed plants. On the other hand, MeJA treatment under heat stress decreased H_2_O_2_ by 44.3%, O^2.−^ by 48.9%, and TBARS by 44.3%, in contrast to heat-stressed plants. However, the collective treatment of MT and MeJA in stressed plants significantly diminished the content of H_2_O_2_ by 55.4%, O^2.−^ by 53.8%, and TBARS by 53.1% relative to values of only heat-stressed plants. Thus, the plants treated with MT or/and MeJA alleviated heat stress by reducing oxidative stress in terms of H_2_O_2_ and TBARS content as well as superoxide radicals.

### Impact of MT or/and MeJA on the antioxidant system under heat stress

**Figure 2:**
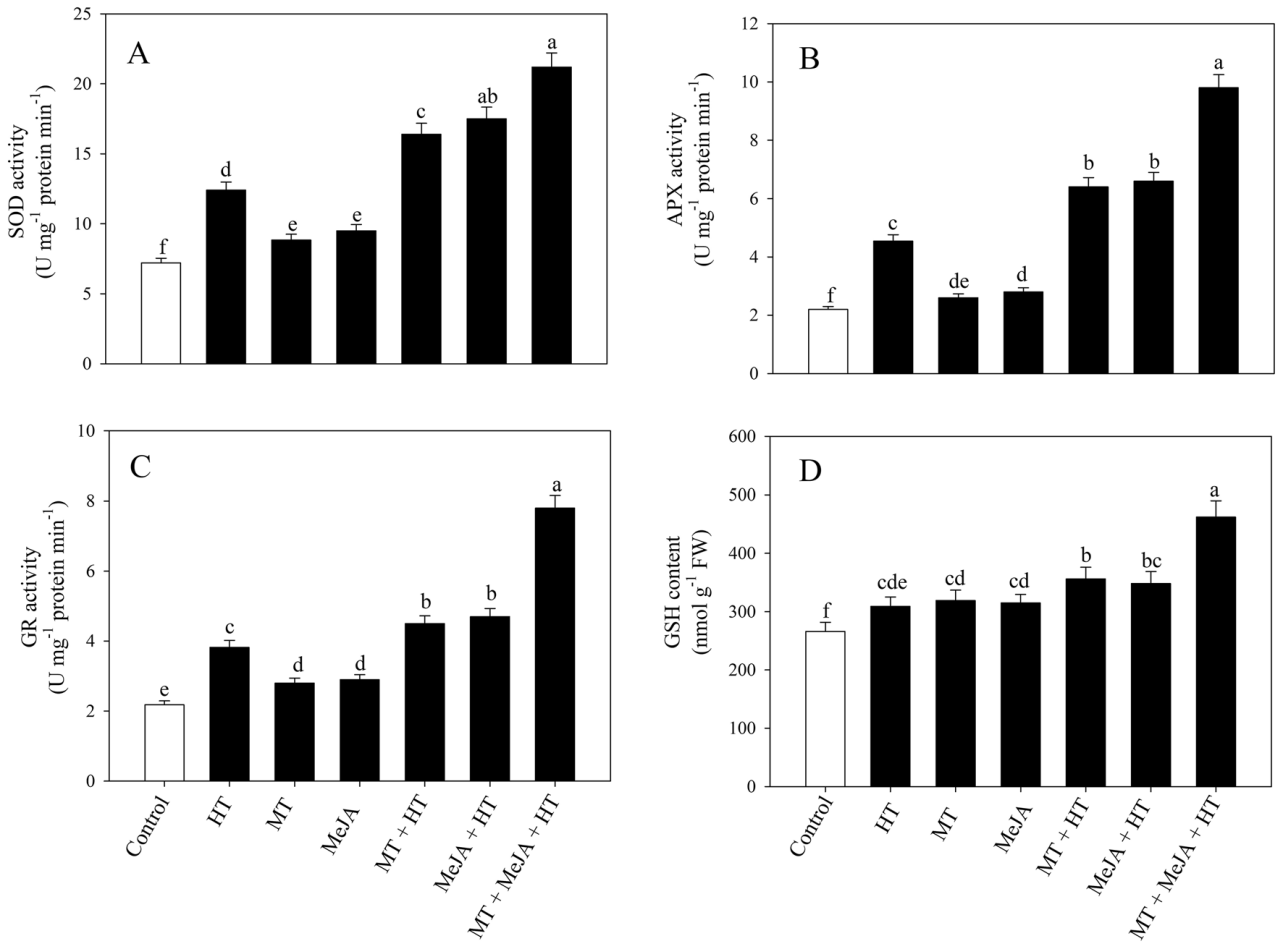
Activity of SOD (**A**), APX (**B**), GR (**C**) and GSH content (**D**) in wheat (*Triticum aestivum* L.) leaves at 30 DAS. Plants were grown with or without high temperature stress (40 °C for 6 h per day for 15 days), and the foliage was treated with 100 µM MT and/or 10 µM MeJA at 20 DAS. Data followed by the same letter are not significantly different from the LSD test at *p* < 0.05. DAS, days after sowing; DAS, days after sowing; HT, heat stress; MT, melatonin; MeJA, methyl jasmonate.

The effect of MT and MeJA on oxidative stress regulation was investigated by measuring the activity of SOD, APX, glutathione reductase (GR), and GSH concentration (Fig. 2A-D). A substantial improvement in the antioxidant enzyme activity and the content of GSH was recorded in all treatments compared to the untreated plants. The enhancement in SOD activity by 72.2%, APX by 106.3%, and GR by 75.2% as well as the content of GSH by 16.1% were recorded under heat-stressed plants in proportionate to the control plants. The solitary supplementation of MT and MeJA under stressed conditions further enhanced the activity of SOD by (32.1% and 41.2%), APX by (40.9% and 45.3%), GR by (178% and 23%) and GSH content by (15.2% and 12.6%), respectively, in contrast to only heat-stressed plants. Nevertheless, the greatest increment in the antioxidants activity was recorded in the heat-stressed plants treated concurrently with MT and MeJA with an increase in the activity of 70.9% for SOD, 115.8% for APX, 104.2% for GR as well as 49.5% for GSH content, relative to heat-stressed plants. These findings suggest that MT and MeJA lowered heat-induced oxidative damage by enhancing the antioxidant system, leading to a reduction in ROS, and were able to restore the disturbed cellular membrane.

### Impact of MT or/and MeJA on the activity of ACS, ethylene production, and related gene expression

**Figure 3:**
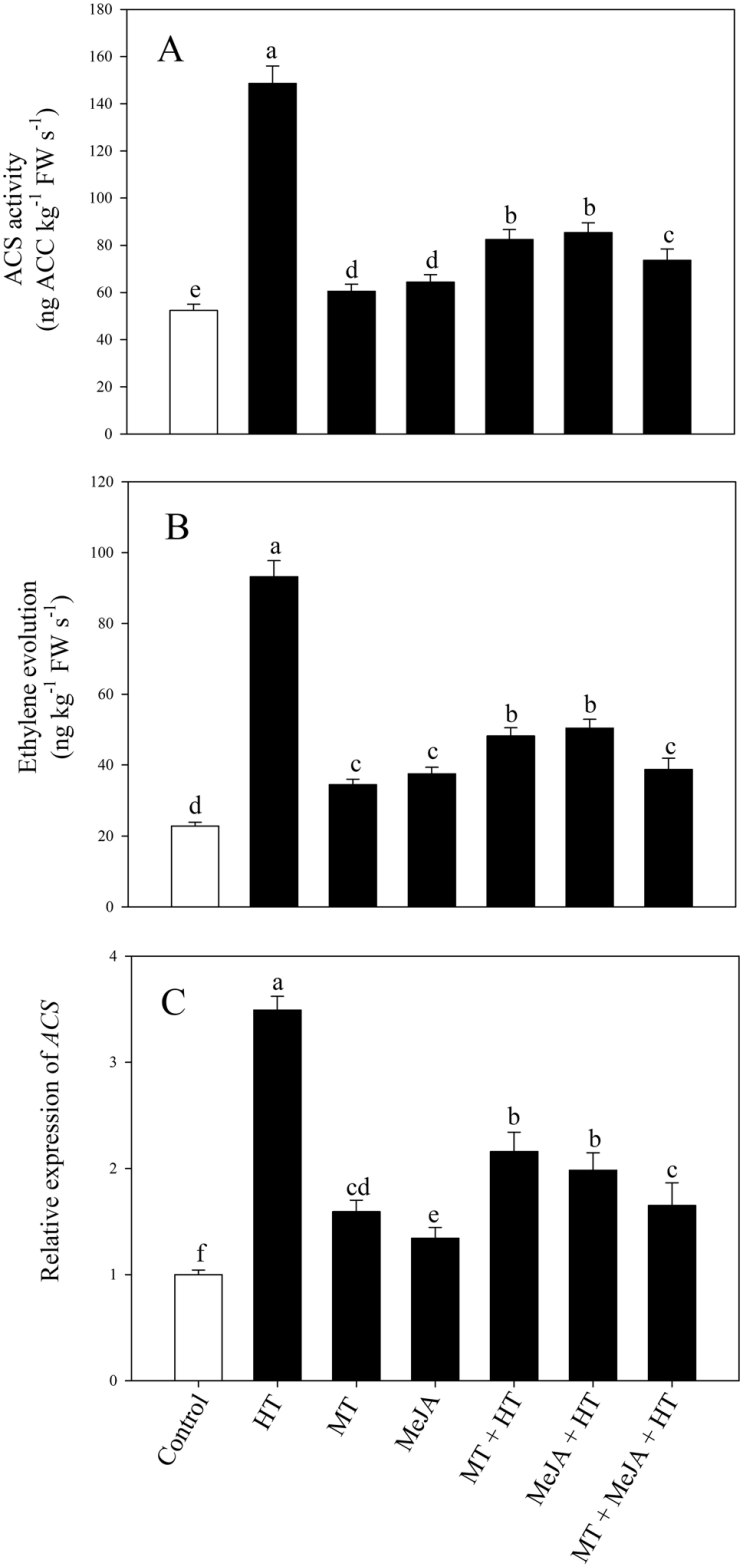
ACS activity (**A**), ethylene evolution (**B**) and relative expression of ACS (**C**) in wheat (*Triticum aestivum* L.) leaves at 30 DAS. Plants were grown with or without high temperature stress (40 °C for 6 h per day for 15 days), and the foliage was treated with 100 µM MT and/or 10 µM MeJA at 20 DAS. Data followed by the same letter are not significantly different from the LSD test at *p* < 0.05. DAS, days after sowing; HT, heat stress; MT, melatonin; MeJA, methyl jasmonate.

High temperature significantly induced the activity of ACS and ethylene generation by 183.5% and 308.6%, respectively, relative to the control plants (Fig. 3A,B). Under unstressed conditions, an increment of (15.4% and 22.9%) in ACS activity and (50.8% and 64.4%) in ethylene production was observed on the sole application of MT and MeJA, respectively, corresponding to the values of control plants. However, the application of MT and MeJA under heat-stressed conditions decreased ACS activity by (44.4% and 42.5%), and ethylene generation by (48.2% and 45.9%), respectively, with respect to only heat-stressed. The combined application of MT and MeJA under heat-stressed conditions reduced the ACS activity by 50.4% and the production of ethylene by 58.4%, respectively, in contrast to only heat-stressed plants. The effect of heat stress and MT and/or MeJA supplementation on the gene expression of the ethylene biosynthesis enzymes ACS was notable. Heat stress considerably enhanced the expression of ACS relative to the untreated plants. The discrete treatment of MT and MeJA to heat-stressed plants exhibited a significant reduction in the ACS expression level relative to only heat-stressed plants. Nevertheless, the maximum reduction in ACS expression level was observed under the collaborated treatment of MT and MeJA in heat-stressed plants (Fig. 3C). Both MT and MeJA regulate the heat stress-induced stress ethylene and bring it to the optimum level via modulating the expression and activity of ACS.

### Influence of MT or/and MeJA on their endogenous content under heat stress

**Figure 4:**
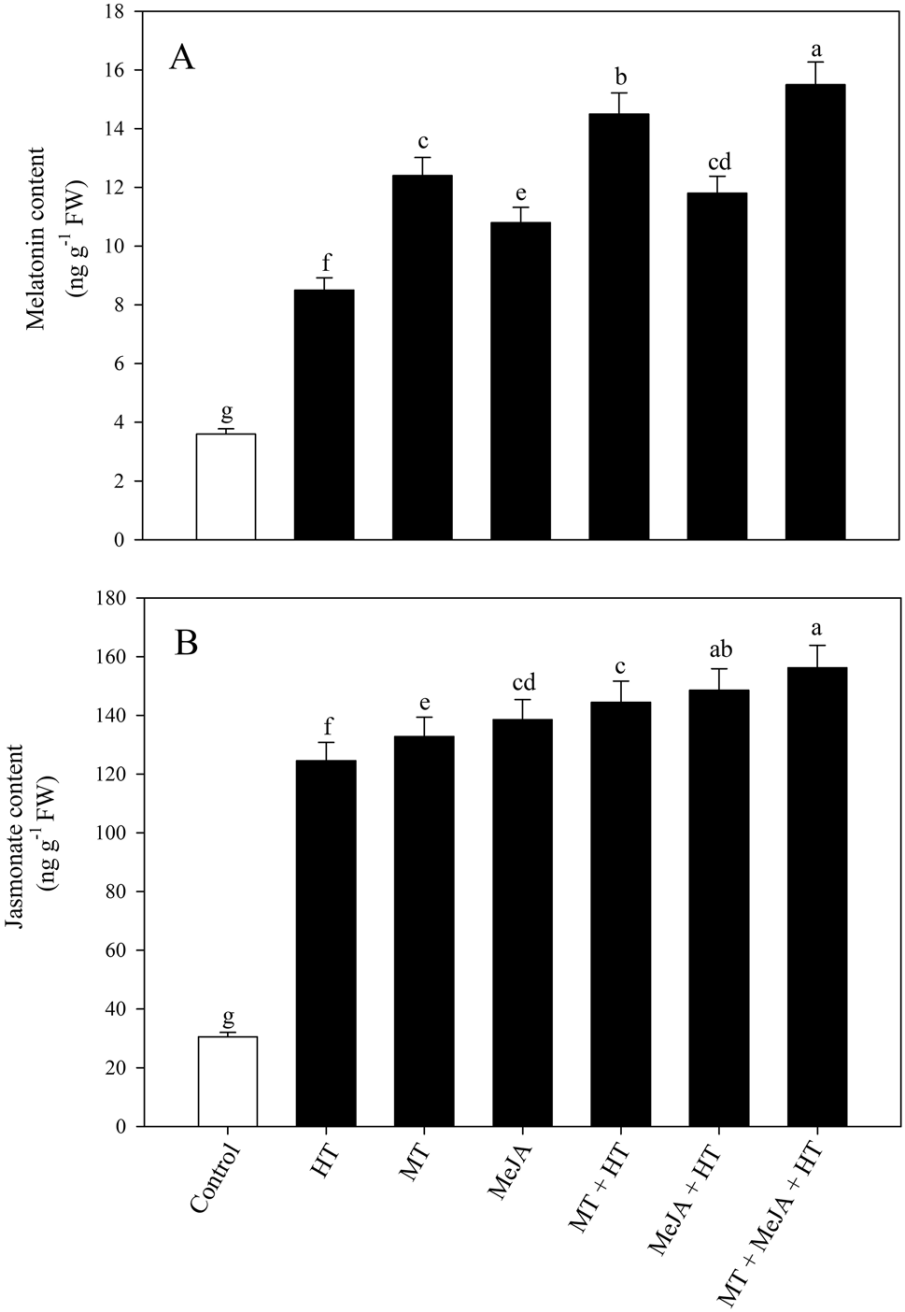
Content of MT (**A**) and JA (**B**) in wheat (*Triticum aestivum* L.) leaves at 30 DAS. Plants were grown with or without high temperature stress (40 °C for 6 h per day for 15 days), and the foliage was treated with 100 µM MT and/or 10 µM MeJA at 20 DAS. Data followed by the same letter are not significantly different from the LSD test at p < 0.05. DAS, days after sowing; HT, heat stress; MT, melatonin; MeJA, methyl jasmonate.

Heat exposure was found to improve the MT content in plants by 136.1% proportionate to the control (Fig. 4A). The plants individually treated with MT and MeJA under heat stress showed enhancement of 70.5% and 38.8%, respectively, in MT content compared with the only heat-stressed plants. However, the highest increase of 82.3% in endogenous MT content was observed in plants with combined treatment of MT + MeJA under heat stress. Similarly, heat stress raised the endogenous MeJA content by 308% relative to the untreated plants (Fig. 4B). The separate treatment of MT and MeJA under stressed and unstressed conditions increased the MeJA content in plants. Nevertheless, the combined treatment of MT plus MeJA under high-temperature stress enhanced the endogenous MeJA concentration by 25.3%, with respect to only the heat stress plant. These findings suggest that MT and MeJA assisted in lowering heat-induced oxidative damage by increasing the endogenous accumulation of MT and MeJA, leading to a reduction in ROS.

### Effect of MT or/and MeJA on photosynthetic and growth attributes

To investigate the efficacy of MT and MeJA on photosynthesis, we examined various photosynthesis-related attributes. Under heat stress, net photosynthesis (Pn), stomatal conductance (Gs), intercellular CO_2_ concentration (Ci), maximum PSII efficiency, and activity of Rubisco enzyme were reduced by 40.6%, 41.6%, 37.8%, 42.1%, and 48.5%, respectively, in contrast to the control. Solitary application of MT and MeJA under normal conditions enhanced the photosynthetic attributes in contrast to the untreated plants. However, under heat stress, the sole administration of MT and MeJA enhanced Pn by (68.4% and 52.6%), Gs by (60.5% and 57.1%), Ci by (51.7% and 47.7%), maximum PSII efficiency of (61.3% and 54.5%), and Rubisco activity by (87.1% and 77.9%), respectively, with respect to only heat-stressed sample. However, the collaborated supply of MT and MeJA under heat stress revealed a maximum increase in the aforementioned photosynthetic attributes by 75%, 11.7%, 12.1%, 5.2%, and 18.9%, respectively, contrary to the control plants, thus reversing the detrimental effect of heat stress. To assess how the MT and MeJA treatment influenced the growth of plant under stress, leaf area and plant dry mass was determined. Heat stress considerably decreased the leaf area by 38.5% and plant dry mass by 38.2% compared to the untreated plants. The plants subjected to the sole application of MT and MeJA under unstressed and stressed conditions enhanced the leaf area and plant dry mass (Table 1). The combined treatment of MT + MeJA under stress was most successful in mitigating heat stress-induced deficits in plant growth, increasing leaf area by 21.9% and plant dry mass by 62.9%, relative to control plants.

**Table 1:** Net photosynthesis, stomatal conductance, intercellular CO_2_ concentration, maximum efficiency of PSII, ribulose 1, 5 bisphosphate carboxylase/oxygenase (Rubisco) activity, leaf area and plant dry mass of wheat (*Triticum aestivum* L.) leaves treated with 100 µM MT and/or 10 µM MeJA in the presence (40 °C) or absence (28 °C) of heat stress at 30 DAS. Data are presented as treatment mean ± SE (n = 4). Data followed by the same letter are significantly different at *p* < 0.05. DAS, days after sowing; HT, heat stress; MeJA, methyl jasmonate.

Treatments	Net photosynthesis (µmol CO_2_ m^−2^ s^−1^)	Stomatal conductance (mmol CO_2_ m^−2^ s^−1^)	Intercellular CO_2_ concentration (µmol CO_2_ mol^−1^)	Maximum quantum yield efficiency of PSII	Rubisco activity (µmol CO_2_ mg^−1^ protein min^−1^)	Leaf area (cm^2^ plant^−1^)	Plant dry mass (g plant^−1^)
Control	12.8 ± 0.58^c^	408 ± 18.8^bc^	314 ± 14.8^bc^	0.76 ± 0.04^bc^	42.4 ± 2.1^bc^	33.2 ± 1.5^b^	0.89 ± 0.04d
HT	7.6 ± 0.34^d^	238 ± 11.5^e^	195 ± 8.8^e^	0.44 ± 0.02^e^	21.8 ± 1.1^e^	20.4 ± 0.9^d^	0.55 ± 0.02f
MT	17.2 ± 0.85^b^	436 ± 20.8^ab^	336 ± 15.8^ab^	0.86 ± 0.04^a^	45.5 ± 2.2^b^	35.5 ± 1.7^b^	1.12 ± 0.05b
MeJA	16.6 ± 0.78^b^	428 ± 19.8^ab^	328 ± 15.8^ab^	0.84 ± 0.04^a^	43.5 ± 0.8^bc^	34.2 ± 1.7^b^	1.0 ± 0.05c
MT + HT	12.8 ± 0.64^c^	382 ± 18.4^ cd^	296 ± 14.2^ cd^	0.71 ± 0.03^ cd^	40.8 ± 1.9^ cd^	29.5 ± 1.3c	0.76 ± 0.04e
MeJA + HT	11.6 ± 0.54^c^	374 ± 17.8^d^	288 ± 13.8^d^	0.68 ± 0.03^d^	38.8 ± 1.8^d^	28.7 ± 1.4c	0.68 ± 0.03e
MT + MeJA + HT	22.4 ± 1.1^a^	456 ± 19.5^a^	352 ± 14.4^a^	0.8 ± 0.04^ab^	50.4 ± 2.5^a^	40.5 ± 1.9a	1.45 ± 0.07a

**Figure 5:**
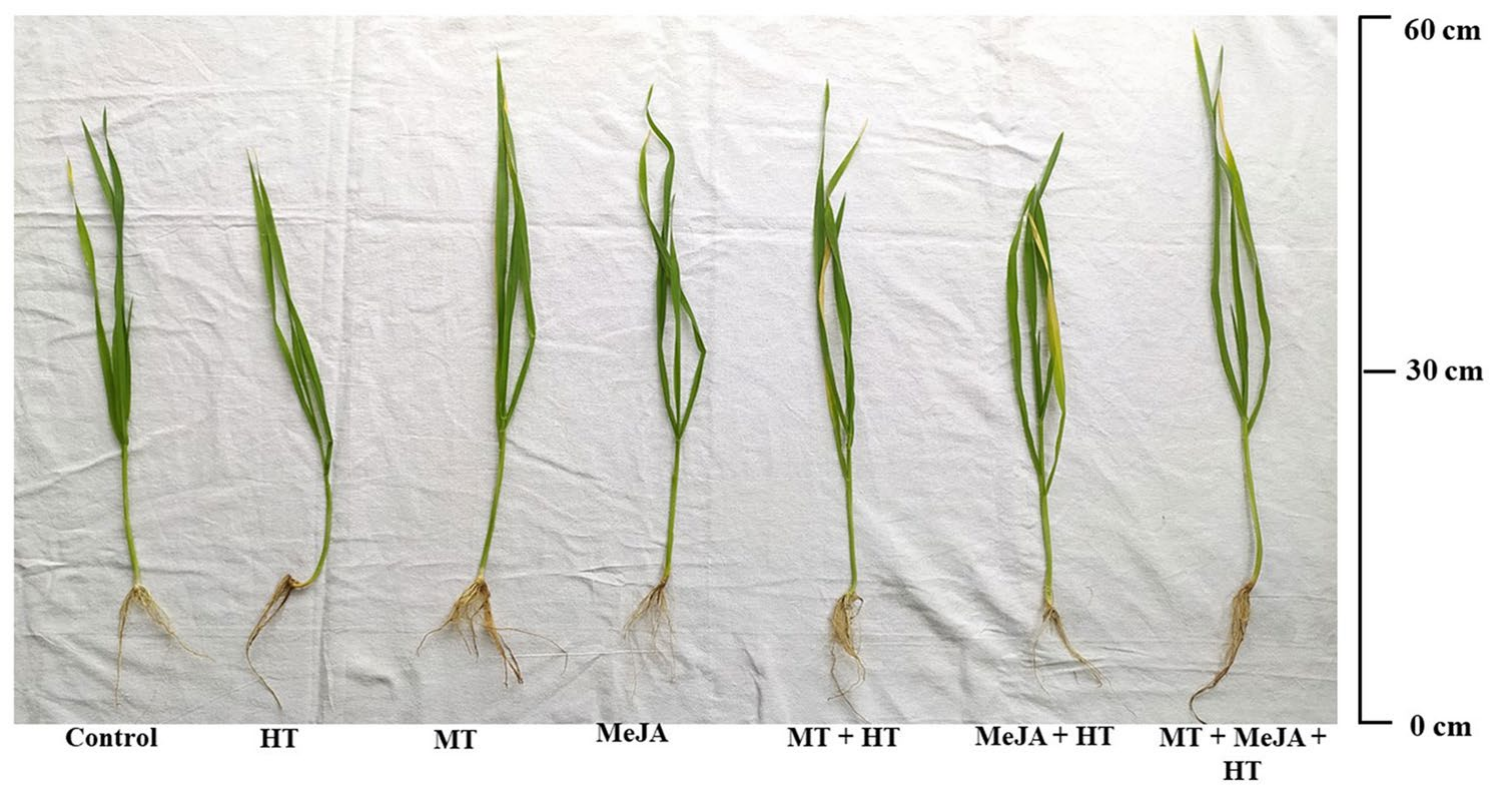
The effect on the morphology of wheat (*Triticum aestivum* L.) under control, heat stress (HT), 100 µM MT, 10 µM MeJA treatment and their combinations at 30 DAS. DAS, days after sowing; HT, heat stress; MT, melatonin; MeJA, methyl jasmonate.

The independent effect of MT and MeJA without or without heat stress is reflected in morphology of plants as shown in Fig. 5. MT and MeJA have the ability to diminish the negative ramifications of heat stress on photosynthesis and the growth of plants (Fig. 5).

### The impact of MT or/and MeJA on the content of S, Cys, and the activity of ATP-sulfurylase (ATP-S) and SAT Enzymes

Heat stress remarkably reduced sulfur content whereas enhanced, Cys content, and activity of ATP-S and SAT relative to the control plants. Application of 100 µM MT under heat-stress circumstances improved S content by 52.6% compared to heat-stressed plants. Similarly, under heat stress, the 10 µM MeJA application enhanced S content by 42.1%, relative to only heat-stressed plants. The collaborative supplementation of MT and MeJA was found to maximally reverse the heat-stressed induced reduction of S content, showing increments of 73.6% in S content compared to heat-stressed plants. The content of Cys and activity of ATP-S and SAT showed enhancement in their values under all treatments. The individual application of 100 µM MT and 10 µM MeJA under heat-stress conditions enhanced the Cys content by (7.5% and 4%), ATP-S activity by (3.1% and 6.25%), and SAT activity by (11.5% and 9.5%) relative to only heat-stressed plants. Nevertheless, the collective treatments of MT and MeJA under heat-stress conditions displayed maximum Cys content of 77.3%, ATP-S activity of 85.1%, and SAT activity of 93%, relative to heat-stressed plants (Table 2). MT and MeJA supplementation increased S-assimilation in terms of S and Cys content, as well as increased the activity of ATP-S and SAT enzymes (Table 2).

**Table 2:** Sulfur content, cysteine content, ATP-sulfurylase (ATP-S) and serine acetyl transferase (SAT) activity of wheat (*Triticum aestivum* L.) leaves treated with 100 µM MT and/or 10 µM MeJA in the presence (40 °C) or absence (25 °C) of heat stress at 30 DAS. Data are presented as treatment mean ± SE (n = 4). Data followed by the same letter are significantly different at *p* < 0.05. DAS, days after sowing; HT, heat stress; MeJA, methyl jasmonate.

Treatments	Sulfur content (mg g^−1^ DW)	Cysteine content ( mg g^−1^ DW)	ATP-S activity (µmol g^−1^ protein s^−1^)	SAT activity (µmol g^−1^ protein s^−1^)
Control	5.2 ± 0.3c	19.2 ± 0.5e	1.72 ± 0.119d	1.9 ± 0.06f
HT	3.8 ± 0.245d	32.6 ± 1.7 cd	2.56 ± 0.136c	2.52 ± 0.16e
MT	6.4 ± 0.4ab	34.9 ± 1.9c	3.11 ± 0.179b	2.8 ± 0.17c
MeJA	6.2 ± 0.378ab	33.9 ± 1.9c	2.88 ± 0.181bc	2.7 ± 0.17 cd
MT + HT	5.8 ± 0.39bc	46.6 ± 2.5b	2.64 ± 0.149c	3.63 ± 0.17b
MeJA + HT	5.4 ± 0.308c	45.4 ± 2.5b	2.72 ± 0.152c	3.67 ± 0.17b
MT + MeJA + HT	6.6 ± 0.42a	57.8 ± 3.7a	4.74 ± 0.336a	4.86 ± 0.27a

### Impact of MT and/or MeJA on the expression of psbA, psbB, and GR

**Figure 6:**
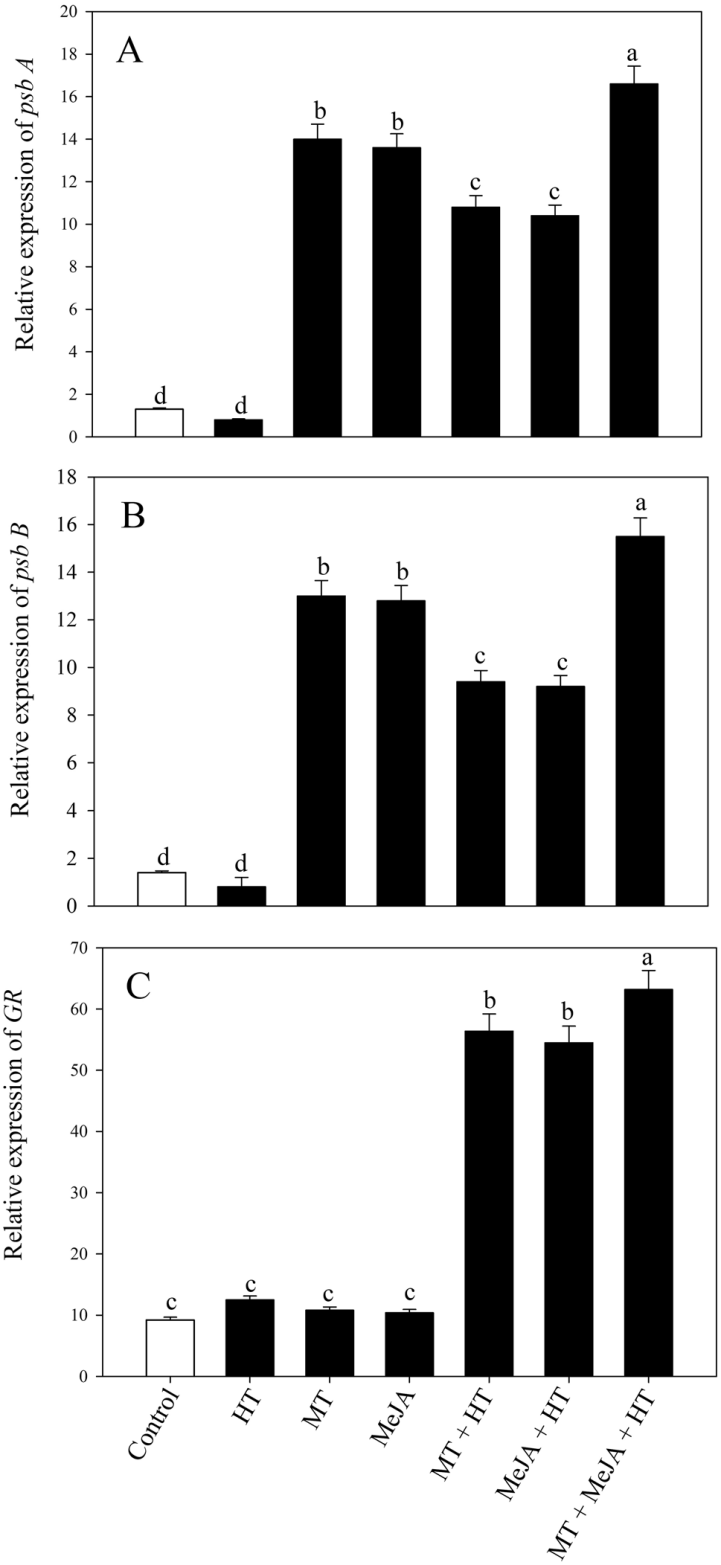
Relative expression of *psb*A (**A**), *psb*B (**B**) and *GR* (**C**) in wheat (*Triticum aestivum* L.) leaves at 30 DAS. Plants were grown with or without high temperature stress (40 °C for 6 h per day for 15 days), and the foliage was treated with 100 µM MT and/or 10 µM MeJA at 20 DAS. Data followed by the same letter are not significantly different from the LSD test at p < 0.05. DAS, days after sowing; HT, heat stress; MT, melatonin; MeJA, methyl jasmonate.

To gain a thorough understanding of photosynthetic mechanisms mediated by MT and MeJA, we examined the expression of the PSII gene *psb*A (D1 protein) and *psb*B (CP47 protein) under different treatments (Fig. 6A,B). Heat stress significantly downregulated the expression of *psb*A and *psb*B by 38.3% and 42.7%, respectively, relative to the control. However, the individual administration of MT and MeJA, both under stressed and unstressed conditions, significantly enhanced the expression of both genes. The collective treatment of MT and MeJA under a heat-stressed plant exhibited a maximum increase in the relative expression level of *psb*A and *psb*B in contrast to the control plants (Fig. 6).

The expression level of *GR* in plants grown under stressed and unstressed conditions was also analyzed (Fig. 6C). The result manifested that heat stress substantially up-regulated the expression of *GR* by 35.8% with respect to the control plants. Administration of 100 µM MT and 10 µM MeJA under optimal and stressed conditions increased *GR* expression level, relative to the control plants. However, under combined application of MT + MeJA under stressed conditions, exhibited the highest expression level of 405.6% relative to only stressed plants. These findings manifested that MT and MeJA treatment increased expression level of *psbA*, *psbB* and *GR*.

### Principal component analysis

**Figure 7:**
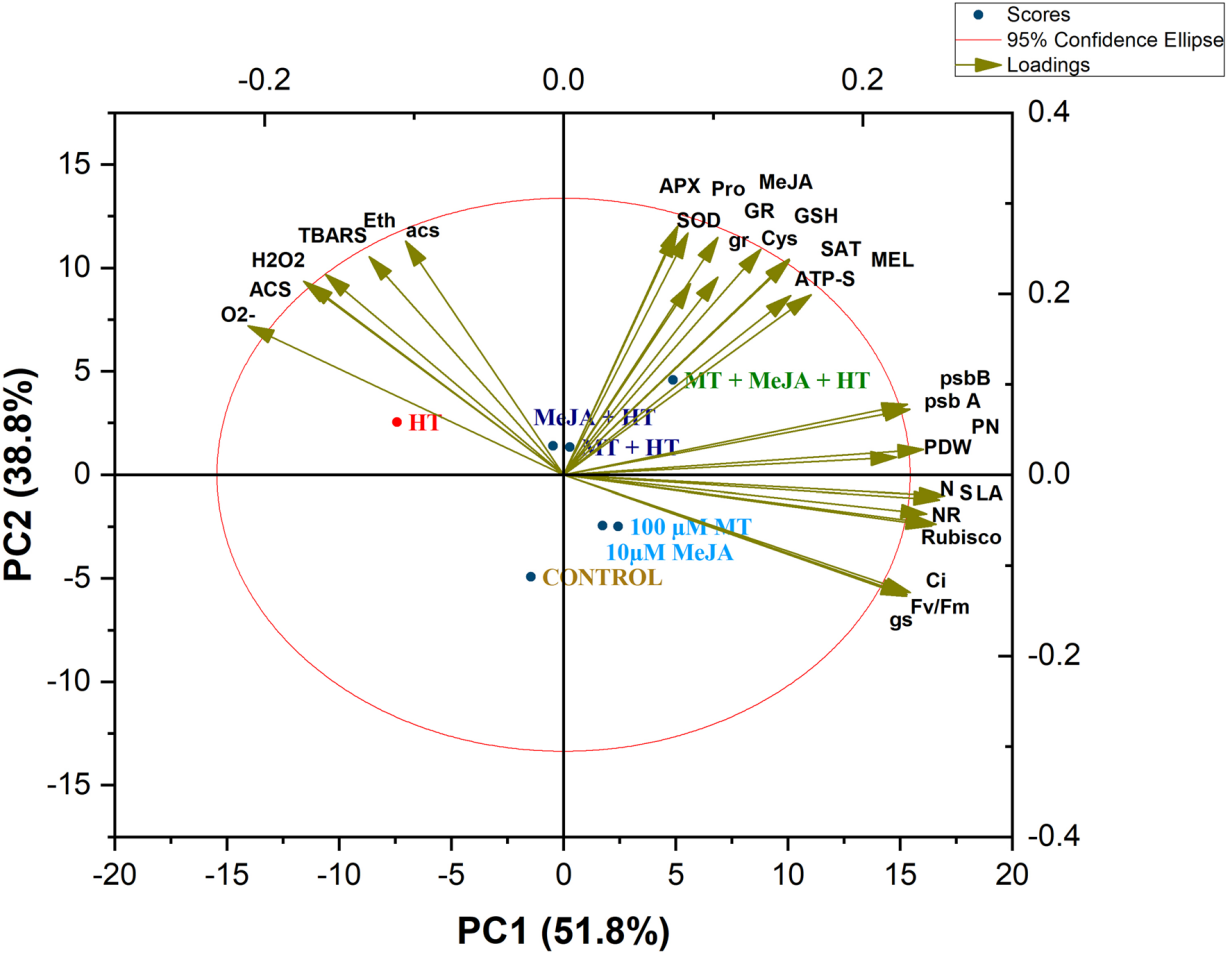
Biplots of principal component analysis (PCA) represent the relationship among different variables and treatments in wheat (*Triticum aestivum* L. cv. WH-711) treated with with/without high temperature and 100 µM MT and/or 10 µM MeJA. The variables included net photosynthesis (Pn), intercellular CO_2_ concentration (Ci), stomatal conductance (Gs), Rubisco activity, maximal PSII efficiency (Fv/Fm), ATP-sulfurylase (ATP-S), serine acetyl transferase (SAT), sulfur content (S), plant dry weight (DW), leaf area (LA), superoxide dismutase (SOD), ascorbate peroxidase (APX), glutathione reductase (GR), reduced glutathione (GSH), melatonin (MT), methyl jasmonate (MeJA), hydrogen peroxide (H_2_O_2_) content, superoxide content (O^2−^), thiobarbituric acid reactive sub-stances (TBARS) content.

A PCA was performed to identify the extent of the variability in data as well as the correlation between the different treatments and attributes (Fig. 7). PC1 and PC2, represent the two most significant components of PCA, which accounted for 90.6% of the variability in data resulting from various treatments (Fig. 7). PC1, represented 51.8% of the total variance, whereas, PC2, contributed 38.8% of the variance. The biplot was divided into three clusters. The oxidative stress biomarkers such as O^2.−^, H_2_O_2_, and TBARS and ACS activity, ethylene evolution, and relative expression of ACS were clustered together. On the other hand, antioxidants were clustered along with MT and MeJA content and S-assimilation characteristics. Growth and photosynthetic attributes were clustered together. The loading plot revealed a positive correlation between plant growth, photosynthetic attributes, chlorophyll fluorescence; GR, SOD, ATP-S, and SAT activity; and GSH expression levels (Fig. 7). Plant growth and photosynthetic characteristics, as well as oxidative stress parameters, were all negatively associated with each other, and antioxidants in the middle, indicating a function in heat stress mitigation. Furthermore, endogenous MT and MeJA are positively correlated with both enzymatic and non-enzymatic antioxidants. Amongst the different treatments, heat stress was adjacent to oxidative stress, however, MT + MeJA with heat stress had a substantial relationship with antioxidants, showing the function of the MT + MeJA with heat stress treatment in alleviating heat stress (Fig. 7). On the other hand, MT and MeJA treatments were close to growth and photosynthetic parameters (Fig. 7).

### Influence of MT biosynthesis inhibitor, p-chlorophenyl alanine (CPA) on Pn, S-assimilation, GSH content, and plant dry mass under heat stress

Supplementation of MT and MeJA lowered the repercussions of heat stress via an increase in GSH synthesis. The effect of MT-induced GSH production and subsequent alleviation of heat stress was not evident when plants received MT biosynthesis inhibitor (CPA). This was substantiated by the supplementation of CPA to the plants and observed the photosynthetic and growth parameters relative to the heat-stressed plants. Plants treated with CPA exhibited reduced GSH content in contrast to the heat-exposed plants. However, supplementation of CPA to heat-stressed plants reduces GSH concentration with the maximum reduction in photosynthetic and growth of plants with respect to the other treatments. Plants treated with MT and/or MeJA under heat stress and CPA showed a greater decline in photosynthetic and growth performance than plants treated with MT and/or MeJA in the presence of heat stress (Table 3).

**Table 3:** Sulfur content, ATP-S activity, GSH content, net photosynthesis and plant dry mass of wheat (*Triticum aestivum* L.) treated with 100 µM CPA, 100 µM MT plus 10 µM MeJA or/and 100 µM CPA in the presence (40 °C) or absence (28 °C) of heat stress at 30 DAS. Data are presented as treatment mean ± SE (n = 4). Data followed by the same letter are significantly different at *p* < 0.05. DAS, days after sowing; CPA, *p*-chlorophenyl alanine; HT, heat stress; MeJA, methyl jasmonate.

Treatments	Sulfur content (mg g^−1^ DW)	ATP-S activity (µmol g^−1^ protein s^−1^)	GSH content (nmol g^−1^ FW)	Net photosynthesis (µmol CO_2_ m^−2^ s-^1^)	Plant dry mass (g plant^−1^)
Control	5.2 ± 0.30c	1.72 ± 0.12e	266 ± 15.7e	12.8 ± 1.15c	0.89 ± 0.044c
HT	3.8 ± 0.24de	2.56 ± 0.13d	309 ± 16.1c	8.2 ± 0.79e	0.55 ± 0.025de
CPA	4.2 ± 0.20d	1.34 ± 0.70f	248 ± 14.6f	11.2 ± 0.95 cd	0.68 ± 0.034d
MT + MeJA + HT	6.6 ± 0.42ab	4.74 ± 0.34a	348 ± 20.6a	19.4 ± 1.4a	1.45 ± 0.072a
MT + MeJA + HT + CPA	3.4 ± 0.17f	4.22 ± 0.2b	326 ± 18.4b	6.7 ± 0.66f	0.43 ± 0.021f

## Discussion

Heat is a detrimental abiotic stressor that poses threats to plants and hinders plant growth and development, and results in significant agricultural output losses ^4^. Understanding the plant responses to heat stress is imperative for developing heat-resistant plants. The MT as an indoleamine, pleiotropic, ubiquitous, and amphiphilic molecule, and JAs are growth regulators that have been involved in alleviating the negative impact of various environmental stress factors such as cold, salinity, drought, and high-temperature stress ^8,21^. A recently discovered plant growth regulator, MT has been demonstrated to have a multifaceted function in plant growth and development ^8,22^. It significantly contributes to the removal of excess ROS and protects plants from oxidative stress by boosting the activity of enzymatic and non-enzymatic antioxidants ^8^. Due to its ease of transport between cell compartments, MT has a stronger antioxidant capacity than non-enzymatic antioxidants, ascorbates, and tocopherols ^23^. The earlier research has demonstrated that an increase in MeJA levels results in a JA-dependent defensive response that improves secondary metabolites ^24^. In an injured plant, the level of MeJA rises noticeably, promoting the biosynthesis of several stress-related molecules and inducing the expression of several stress tolerance genes, thereby boosting the plant's resistance to environmental stress ^25^. Application of MT to plants altered the expression of genes related to redox components and antioxidants, as a result it detoxified over accumulated ROS and reactive nitrogen species (RNS) in harsh environments. This ultimately improved the growth and development of the plants receiving MT ^7,26^. In the present study, we observed that reduced photosynthesis and growth due to heat stress was recovered by the MT and MeJA supplementation. Furthermore, we found that MT and MeJA regulated S-assimilation, which influenced antioxidant metabolism and ethylene production to minimize the heat stress effects. The optimized ethylene levels participated in heat stress resistance by upregulating the enzymatic and non-enzymatic antioxidants and reducing the glucose sensitivity. Ethylene has been shown to regulate S-assimilation, ascorbate–glutathione cycle and production of GSH to limit the oxidative stress under heat stress through enzymatic and non-enzymatic antioxidants. Under heat stress, the controlled formation of ethylene termed as stress ethylene negatively impacted plant performance. However, the optimized ethylene formation with MT and MeJA treatment positively influenced the photosynthesis.

In the present study, heat stress had a detrimental impact on photosynthesis and growth, which may be related to a high concentration of oxidative stress indicators such as H_2_O_2_ and TBARS content, and superoxide radicals. Heat stress conspicuously decreased photosynthesis, through changes in stomatal conductance and intercellular CO_2_ concentration High-temperature stress has been seen to alter pigment levels, photosynthesis, and growth in a different types of plants ^4,27,28^. Additionally, prior studies have revealed that extreme heat stress can cause cellular damage, cell death, and a decline in the plant’s total dry mass ^29^. Earlier studies have shown that both MT and MeJA act as active plant growth regulators, involved in the heat tolerance of wheat plants. In order to find more about the mechanisms induced by MT and MeJA for heat tolerance, their integrated effect on S-assimilation, antioxidant system and ethylene production was studied. The findings suggest that MT and MeJA upregulated S-assimilation for the higher synthesis of antioxidants, such as GSH, and also reduced excess ethylene production under heat stress, more conspicuously when they were applied together. The higher GSH production by MT and MeJA was linked to increased turnover of the ascorbate–glutathione cycle with the higher activity of APX and GR for the regeneration of GSH. Both MT and MeJA are important signaling molecules, and they work synergistically to regulate each other’s behaviour, which helps improve growth and photosynthetic performance of the plant. Exogenously applied MT and MeJA have been shown to promote cold-tolerance in tomatoes by reducing oxidative stress indicators in terms of H_2_O_2_ and TBARS and electrolyte leakage as well as the increase in MT and MeJA content ^22^. Furthermore, according to a study by Ding et al. ^22^, qRT-PCR assay revealed that MeJA enhanced the expression of two genes involved in MT biosynthesis (*SlSNAT and SlAMST*), which were suppressed under cold stress. However, MeJA decreased the expression of the JA signaling regulator *SlMYC2*. These findings collectively imply that JA and MT cooperate in cold tolerance and create a positive feedback loop that amplifies tomato plants' cold responses ^22^. Awasthi et al. ^30^ showed that the excess of ROS seriously harmed lipids and proteins, which was a major factor in the reduction of plant development. However, combining heat stress with MT and MeJA drastically restricted ROS production and caused a significant fall in cell membrane damage. Further, studies have revealed that applications of MT and MeJA in optimum concentration have the potential to limit the content of ROS in plants under abiotic stress ^8,31^. The treatment of MT has a crucial role in boosting endogenous MT quantification, which aids plants in avoiding oxidative stress caused by abiotic stress by lowering levels of ion leakage, H_2_O_2_, O^2.−^, and TBARS ^8,22^. In addition, it was noted that MT and MeJA-treated wheat seedlings exhibited higher antioxidant activity and their expressions, as well as accumulation of osmoprotectants (proline), suggesting that MT could improve heat tolerance of plant by scavenging excess ROS and attuning ROS balance ^8,31,32^. Studies by Su et al. ^32^ reported that MeJA alleviated heat stress tolerance by decreasing lipid peroxidation and up-regulating the activity of SOD, APX, and GR and GSH content, thus enhancing the scavenging ability of ROS. Exogenous application of MeJA enhances the transcript levels of various genes involved in JA biosynthetic pathways such as (*LpLOX2*, *LpAOC*, *LpOPR3*, and *LpJMT*), which could be directly related to increased JA content ^33^. Reports have demonstrated that JA is a signaling molecule that controls the expression of genes involved in defense against a variety of environmental stressors ^34^. Wasternack ^35^ reported that JA controls the expression of genes involved in defense through its signaling pathways, which are activated by JA compounds. Additionally, the biosynthesis of JA is regulated by positive feedback, which amplifies the signal and helps to ensure an appropriate response to environmental stressors. A study by Mao et al. ^36^ demonstrated that exogenous MeJA treatment dramatically enhanced LOX2 in the JA biosynthetic pathway of *Arabidopsis thaliana* seedlings. In our study, results showed that individual as well as combined application of MT and MeJA could increase the photosynthetic and growth characteristics of plants, as well as the increase in the activity of expression of antioxidant enzymes and genes regulating PSII activity. Furthermore, MT and MeJA content was also increased under high-temperature stress, indicating these compounds play a key role in the plant's ability to respond favorably to heat stress. Several studies have demonstrated that endogenous jasmonates concentration swiftly and enormously accumulates in plants under stressful situations ^37^ and that the endogenous content of JA and MeJA steadily rose with the upregulation of critical JA pathway genes ^33^. These findings suggested that the signal route of JAs may be positively regulated by heat stress and exogenous MeJA, increasing the concentration of JAs in leaves ^33^. In stressed plants, JA therapy may cause plants to develop protective mechanisms to counteract the negative effects of stress, such as the synthesis of proteins with specific functions, the induction or activation of associated enzymes, and the production of secondary active compounds, in order to fend off the effects of adversity. Hu et al. ^38,39^ demonstrated that *NAC* genes play an important role in mitigating biotic and abiotic stresses. Previous studies exhibited that NAC proteins had a significant role in the regulation of abiotic stress ^33^. In addition to this, we observed in our study that the expression level of PSII-associated genes such as *psb*A, p*sb*B, and genes responsible for ACS activity and ethylene evolution such as ACS expression was elevated which caused a significant function in the alleviation of heat stress tolerance. According to the previous study, it is well documented that MT treatment boosts the activity of antioxidant enzymes (SOD, APX, GR) and the content of non-enzymatic antioxidants (GSH) and it also upregulates gene expression of an antioxidant such as SOD, APX, and GR by decreasing ROS production by lowering the oxidative stress brought on by alterations in plant growth and development as well as lipid peroxidation ^39,40^. MT has been demonstrated to increase the expression of *CAT 1*, *APX*, and *GR 1* genes in *Solanum lycopersicum* L. ^41^.

The complex of proteins and pigments known as photosystem II is crucial for the reduction of plastoquinone, oxygen evolution, and water splitting. Compared to PS I in chloroplasts, PS II is more sensitive to abiotic stress ^4,42,43^. It has been demonstrated that chlorophyll fluorescence characteristics such as the efficiency of PSII, can accurately indicate the level of stress in plants ^44^. Yin et al. ^45^ observed that salt stress reduced the oxygen-evolving complex's activity and electron transfer in PS II, but this was reversed by melatonin-mediated repair of PS II by preserving the amount of psbA and D1 protein. When exposed to salt stress, melatonin administration increased the expression of photosynthesis-related genes PSI and PSII in soybean plants relative to untreated soybean plants^46^. In the present study, to learn more about how melatonin and methyl jasmonate works to protect wheat plants against heat stress, the expression of the two genes involved in the photosynthetic system *psbA* and *psbB*, which respectively encode the proteins D1 and CP47 was examined. We found that heat stress decreased the expression of *psbA* and *psbB*, but MT and MeJA treatment significantly raised it when compared to controls under normal circumstances. The expression of *psbA* and *psbB* was noticeably elevated in plants that were exposed to MT and heat stress. According to the findings of this study, wheat plants exposed to heat had lower chlorophyll fluorescence characteristics, which in turn caused Pn to drop. This suggests that heat stress-induced ROS generation lowered PSII efficiency and impaired the reaction center's capacity to utilize light energy effectively. Marutani et al. ^47^ demonstrated that extreme heat stress altered the structure of protein complexes degrading the photosystem and activity of their oxygen-evolving complexes, all of which affect the photosystem's capacity to transport electrons. Thus, the administration of MT and MeJA alleviated the photoinhibition and diminished photosynthetic properties induced by heat stress exposure. It was shown from the present study that there was an interactive relationship between the action of MT and MeJA in the regulation of the photosynthetic process through the antioxidant system and S-assimilation under heat stress. The suppression of MT by using its inhibitor CPA along with MeJA, and MT together with MeJA plus heat stress reduced S-assimilation and GSH production, photosynthesis, and growth, suggesting the potential role of MT in MeJA-evoked antioxidant system, S-assimilation, and regulation of photosynthesis, and growth thermotolerance.

To further confirm the role of MT and MeJA in heat stress tolerance, we run our data for principal component analysis. The relation between all paired attributes such as antioxidant metabolism, and their expression showed a positive response with photosynthetic and growth metrics, whereas it was negatively correlated with oxidative stress biomarkers.

Based on our findings, it is inferred that heat stress impairs plant photosynthetic and growth performance. Exogenous MT supplementation optimized and upregulated the performance of antioxidant enzymes, reducing the suppressive effect of heat stress on photosynthetic efficiency. This enhancement in photosynthetic efficiency was connected to the optimization of heat stress-induced toxicity under MT and MeJA supplementation via regulation of glutathione synthesis, which facilitated reduced ROS generation and improved antioxidant enzyme activity. The application of MT biosynthesis inhibitor (CPA) confirmed the involvement of MT and MeJA on photosynthetic performance under heat stress via MT- modulated GSH synthesis. The current work implies that MT and MeJA supplementation in an agricultural setting could be utilized to boost photosynthesis and growth under heat stress.

## Materials and methods

### Plant material, growth conditions, and treatments

Wheat (*Triticum aestivum* L. cv. WH-711) were obtained from the National Seeds Corporation, New Delhi, India). Healthy seeds were sanitized with 0.01% HgCl_2_, washed repeatedly with deionized water, and seeded in 15 cm diameter earthen pots filled with an adequate amount of acid-rinsed sand. The plants were maintained in the experimental area of the Department of Botany, Aligarh Muslim University, Aligarh (India), with day/night temperatures of 25/18 ± 3 °C, 12 h photoperiod (680 µmol m^−2^ s^−1^), and relative humidity of 65 ± 5%. Two plants per pot were maintained and drenched with 300 mL of full-strength Hoagland’s nutrient solution on alternate days.

In the experiment, plants were exposed to 40 °C temperature for six hours per day for 15 days to induce heat stress. Afterward, plants were given a growth period of 5 days under optimal conditions at 28 °C. Control plants were kept at 28 °C throughout the entire 30-day experiment. To investigate the effect of MT and MeJA on heat stress, the plants were treated with 100 µM MT and/or 10 µM MeJA on the foliage of stressed and unstressed plants after 20 days of sowing., concentrations are based on previous findings ^8,15,22^. Additionally, to substantiate the role of MT in MeJA-induced heat stress alleviation, 100 µM p-chlorophenyl alanine (an inhibitor of MT biosynthesis) was added individually or in combination with MT + MeJA with heat stress treatment. The solution containing hormones and inhibitors was sprayed in a volume of 20 mL, and a surfactant teepol was added at 0.5% to improve absorption. The design of the experiment consisted of a completely randomized block with four replicates per treatment (n = 4).

All the methods were carried out in accordance with the the relevant guidelines.

### Measurement of photosynthetic and growth indices

In order to evaluate photosynthetic parameters (Pn, Ci, Gs) in the fully grown top leaves, we used Infrared Gas Analyzer (CID-340, Photosynthesis System, Bio-science). The observations were made between 11:00 a.m. and 12:00 p.m. with an atmospheric CO_2_ concentration of 380 µmol mol^−1^, relative humidity of 70%, an amount of PAR of 580 µmol m^−2^ s^−1^, and a temperature of 26 °C.

The maximal efficiency of photosystem II (PSII), as given by Fv/Fm, was determined using a chlorophyll fluorometer (Junior-PAM, Heinz Walz, GmbH, Germany). The process is detailed in Iqbal et al. ^30^.

Ribulose 1,5-bisphosphate carboxylase/oxygenase (Rubisco, EC 4.1.1.39) activity was spectrophotometrically determined by adopting the method of Usuda ^48^. The details are given in Iqbal et al. ^30^.

Plants were plucked from their pots and cleaned thoroughly to remove any soil debris from the roots in order to measure the growth characteristics. To measure the dry biomass of the plants, they were kept in a hot air oven at 80 °C until a consistent weight is achieved. The leaf area was calculated using a leaf area meter (LA211, Systronics, New Delhi, India).

### Measurement of H_2_O_2_ and TBARS contents

The H_2_O_2_ assay was conducted using the Okuda et al. ^49^ method, which has already been discussed Sehar et al. ^28^. The Supplementary File S1 contains the method's specifics. Fresh leaf tissues (500 mg) were macerated in 200 mM perchloric acid until ice-cold, after which they were spun at 1200 g for 10 min. The supernatant was later neutralised using 4 M KOH. The homogenate was then centrifuged at 500 g for three minutes to remove the insoluble potassium perchlorate. The reaction mixture (1.5 mL) contained 80 µl of 3-methyl-2-benzothiazoline hydrazone, 20 µl of 3-methyl-2-benzothiazoline hydrazone, 400 µl of 12.5 mM 3-(dimethylamino) benzoic acid in 0.375 M phosphate buffer (pH 6.5), and 1 mL of the eluate. At 25 C, the reaction was initiated after peroxidase was introduced. The rise in absorbance was estimated at 590 nm using a spectrophotometer. According to Dhindsa et al. ^50^ explanations, the amount of thiobarbituric acid reactive substances (TBARS) was calculated to measure the degree of lipid peroxidation.

Superoxide (O^2•−)^ production was determined by adopting the method of Elstner and Heupel ^51^. The details of the procedure is given in Khan et al. ^8^.

### Assay of antioxidants enzyme activity and content of GSH

Using a cold mortar and pestle, fresh leaves were homogenized with an extraction buffer comprising 0.05% (v/v) Triton X-100 and 1% (w/v) PVP in potassium-phosphate buffer (100 mM, pH 7.0). After centrifugation, the supernatant was used for the SOD (EC; 1.15.1.1) and GR (EC; 1.6.4.2) enzyme assays. Extraction buffer was used in addition to 2.0 mM ascorbate for the measurement of APX (EC; 1.11.1.11). The activity of SOD was assayed by the method of Beyer and Fridovich ^52^ and Giannopolitis and Ries ^53^. The activity of APX was determined following the method of Nakano and Asada ^54^ by recording the decrease in the absorbance of ascorbate at 290 nm. The activity of GR was determined by the method of Foyer and Halliwell ^55^ by monitoring the glutathione-dependent oxidation of NADPH at 340 nm. The details of the method are given in Supplementary File S1.

Following Griffith's method ^56^ decreased GSH was measured using an enzymatic cycle. In this, in the presence of GR, was successively oxidized by 5,5-dithiobis-2-nitrobenzoic acid (DTNB) and reduced by NADPH. In Fatma et al. ^13^, the specifics for determining GSH are given.

### Quantification of ACS activity and ethylene production

The activity of 1-aminocyclopropane-carboxylic acid-synthase (ACS; EC, 4.4.1.14) was measured by adopting the methods of Avni et al. ^57^ and Woeste et al. ^58^. Leaf tissue (5.0 g) was homogenized in 100 mM HEPES buffer (pH 8.0) containing 4 mM DTT, 2.5 mM pyridoxal phosphate, and 25% PVP. The homogenized material was centrifuged at 12,000 × g for 15 min. One mL of the supernatant was placed in a 30 mL tube and 0.1 mL of 5 mM S-adenosyl methionine (AdoMet) was added and incubated for 2 h at 22 ºC. The ACC formed was determined by its conversion to ethylene by the addition of 0.1 mL of 20 mM HgCl2 followed by the addition of 0.1 mL of a 1:1 ratio of saturated NaOH/NaCl and placed on ice bath for 10 min. In the control set, Ado Met was not added.

The level of ethylene was estimated using a gas chromatograph. The details of the procedure have been given earlier by Fatma et al. ^13^ and presented in Supplementary File S1.

### Estimation of ATP-S and SAT activity, S and Cys content

The methodology of Lappartient and Touraine ^59^ and Kredich and Tomkins ^60^ was used to measure the activity of ATP-S and SAT (serine acetyl transferase). The activity of ATP-S in leaves was measured in vitro by monitoring molybdate-dependent pyrophosphate synthesis.

Quadrupole ICP-MS (model Elan DRC II; PerkinElmer SCIEX Inc.) was used to measure the S content ^13^. To reduce the potential issues brought on by reactive contaminant species that have not been found, high-purity He and H_2_ were used. Leaf Cys content was quantified by following the approach of Gaitonde ^61^.

### Quantification of MT and MeJA content

High-Performance Liquid Chromatography (HPLC) was used to quantify MT. The samples were first homogenized with chloroform at 4 °C in the dark, and the chloroform phase was then purified using a SPE C18 cartridge after centrifugation at 4000×*g* for 5 min. For HPLC analysis, the particles from the evaporated extracts were resuspended in methanol, using an HPLC system with a 5 mm Hypersil ODS column and a fluorescence detector. The mobile phase methanol flow at the rate of 1.0 mL min^−1^. 20 mL of the sample was injected into the system. The excitation wavelength was set to 280 nm and the emission wavelength was set to 348 nm for MT detection.

Following standard protocols with a few minor adjustments, MeJA quantification was carried out ^62^. Shortly after being homogenized in ethyl acetate, leaves were crushed in liquid nitrogen. Shaking occurred overnight at 4 °C with the homogenate. After that, the homogenate underwent a 10-min centrifugation at 18,000*g*. After the pellet had been resuspended in ethyl acetate, the supernatant had been collected. The pellet had then been centrifuged for 10 min at 18,000*g*. With the help of nitrogen gas, the supernatants were combined and dried. The remnant was reinstated in the supernatants were submitted to HPLC analysis after being dissolved in methanol and centrifuged at 18,000*g* for 2 min. A 3.5 mm Agilent ZORBAX XDB column was used for the HPLC study (C18). 0.1 percent formic acid and 0.3 ml per minute of methanol made up the mobile phase. The system received a 20 mL sample injection and the temperature of the column was maintained at 40 °C.

### RNA isolation and cDNA synthesis

Using TRIzol reagent (Ambion, Life Technologies, Austin, TX, USA), total RNA was extracted from leaves of plants following manufacturer’s instructions. The extracted RNA was then quantified using Nanodrop spectrophotometer (Thermo Scitinfic, Walthm, MA, USA). The details of the procedure are described earlier by Gautam et al. ^4^ and presented in Supplementary file 1.

### Quantitative real-time PCR analysis

Real-time PCR (RT-PCR) was performed in a 96-well reaction plate (Roche, Mannheim, Germany) on a thermal cycler (Light cycler 480 II, Roche, Germany). The setup consisted of reaction a mixture (20 µL) of × 10 reaction buffer, 10 µL cDNA template, 1 mM MgCl2, 2 mM dNTPs, 1 µL Sybr green (× 10), 0.35 µM each of forward and reverse primers, and 5 U Taq polymerase. The details of the procedure are given in Gautam et al.^4^ and presented in Supplementary file S1. The data was analyzed as the differential expression of the target gene between the treated sample and the untreated control relative to the internal control.

### Statistical analysis

SPSS 17.0 for windows was used to perform ANOVA for the data evaluation and shown as a mean ± SE (n = 4). At *p* < 0.05, the least significant difference was determined for the significance of the data. Bars with the same letter imply that the means are not substantially different. Origin Pro (v 9.8) for windows was used to do principal component analysis between distinct variables.

## Conclusion

In conclusion, the results demonstrated that MT with MeJA improved photosynthetic and growth responses both under normal and heat-stressed conditions. The heat impacts elicited oxidative stress in plants due to an increase in ROS production. Exogenous MT supplementation effectively alleviated the deleterious repercussions of heat stress; and when combined with MeJA, it significantly mitigated the heat-induced detrimental effects on photosynthesis and plant growth. The stimulating effects of MT and MeJA on PSII photochemistry were manifested through the higher expression of *psb*A and *psb*B. Together, MT plus MeJA treatments were most beneficial in enhancing heat stress tolerance through their influence on antioxidant metabolism, ethylene synthesis, and S-assimilation. There was an interaction of MT and MeJA in eliciting heat tolerance in plants. MeJA-induced response and thermotolerance were regulated by the presence of MT. The study provides an approach for heat stress remediation in wheat plants and elaborates the interaction with ethylene and nutrient and antioxidant metabolism, which can be used for modifying plant genotypes suitable for heat stress tolerance.

## Supplementary Information

Supplementary Information.

## Data Availability

Data supporting the findings of this work are provided in the paper and its Supplementary Information file. All other raw data that support this paper and other findings of this study are available from the corresponding author upon reasonable request. Data and materials will be shared with no restrictions on the availability of raw or processed data via a material transfer agreement.
