# Supplementary material for: Melatonin influences methyl jasmonate-induced protection of photosynthetic activity in wheat plants against heat stress by regulating ethylene-synthesis genes and antioxidant metabolism
Source: Sci Rep. 2023 May 8;13:7468. doi: 10.1038/s41598-023-34682-y (PMC10167371; doi:10.1038/s41598-023-34682-y)
Supplement: Supplementary file 1 — Supplementary Information. [file 41598_2023_34682_MOESM1_ESM.docx]

**Supplementary File S1: Methodology and Table S1**

**Melatonin influences methyl jasmonate-induced protection of photosynthetic activity in wheat plants against heat stress by regulating ethylene-synthesis genes and antioxidant metabolism**

**Zebus Sehar^1^, Mehar Fatma^1^, Sheen Khan^1^, Iqbal R. Mir^1^, Gholamreza Abdi^2^ and Nafees A. Khan^1*^**

^1^Plant Physiology and Biochemistry Laboratory, Department of Botany, Aligarh Muslim University, Aligarh 202002, India

^2^Department of Biotechnology, Persian Gulf Research Institute, Persian Gulf University, Bushehr, Iran

*Corresponding Author: naf9.amu@gmail.com

*Assay of activity of antioxidants enzymes*

Fresh leaves (200 mg) were homogenized with an extraction buffer containing 0.05% (v/v) Triton X-100 and 1% (w/v) PVP in potassium-phosphate buffer (100 mM, pH 7.0) using chilled mortar and pestle. The homogenate was centrifuged at 15,000 x g for 20 min at 4oC. The supernatant obtained after centrifugation was used for the assay of SOD (EC; 1.15.1.1) and GR (EC; 1.6.4.2) enzymes. For the assay of APX (EC; 1.11.1.11), 2.0 mM ascorbate was supplemented with extraction buffer.

Activity of SOD was determined by adopting the methods of Beyer and Fridovich (1987) and Giannopolitis and Ries (1977) by monitoring the inhibition of photochemical reduction of NBT. 5 mL of reaction mixture consisting of 5 mM HEPES (pH 7.6), 50 mM Na2CO3 (pH 10.0), 0.1 mM EDTA, 0.025% (v/v) Triton X-100, 13 mM methionine, 63 mmol NBT and 1.3 mmol of riboflavin. The enzyme extract was Illuminated for 15 min (360 μmol m^2^s^-1^), and a set was not illuminated which acted as a control to correct for the turbidity of background absorbance. One unit of SOD is defined as the amount of enzyme that inhibited the NBT reduction by 50% at 560 nm. Amount of enzyme that inhibited the reduction of NBT by 50 % at 560 nm is equal to one unit of SOD.

The activity of APX was determined following the method of Nakano and Asada (1981) by recording the decrease in the absorbance of ascorbate at 290 nm. A 1.0 mL assay mixture contained 50 mM phosphate buffer (pH 7.0), 0.1 mM EDTA, 0.5 mM ascorbate and 0.1 mM H_2_O_2_, and the enzyme extract. APX activity was calculated by using the extinction coefficient of 2.8 mM^-1^cm^-1^. One unit of the enzyme is the amount necessary to decompose 1 µmol of substrate per min at 25^o^C.

The activity of GR was determined by the method of Foyer and Halliwell (1976) by monitoring the glutathione-dependent oxidation of NADPH at 340 nm. The reaction mixture contained phosphate buffer (25 mM, pH 7.8), 0.5 mM GSSG, 0.2mM NADPH, and the enzyme extract. The activity of GR was calculated by using the extinction coefficient 6.2 mM-1 cm-1. One unit of enzyme is the amount necessary to decompose 1 µmol of NADPH per min at 25^o^C.

*Ethylene evolution*

Ethylene was measured by placing 0.5 g of cut leaf material into 30 mL tubes containing moist paper to minimize evaporation from the tissue and stoppered with secure rubber caps and placed in light for 2 h under the same condition used for plant growth. The 1 mL gas sample of 1 mL was withdrawn from the tubes with a hypodermic syringe and assayed on a Nucon 5700 gas chromatograph (Nucon Engineers Private ltd., New Delhi, India) equipped with a 1.8-m PorapackTM N (80–100 mesh) column (Sigma-Aldrich, St. Louis, MO, USA), a flame ionization detector and data station. Nitrogen was used as carrier gas. The flow rates of nitrogen, hydrogen, and oxygen were 30, 30, and 300 mL min^−1^, respectively. The detector was set at 150 ◦C. Ethylene was identified based on the retention time and quantified by comparison with peaks from standard ethylene concentration.

*RNA Isolation and cDNA Synthesis*

Total RNA was isolated from rice leaves using TRIzol reagent (Ambion, Life Technologies, USA)

according to the manufacturer’s instructions. With the help of a Nanodrop spectrophotometer (Thermo Scientific, USA), the extracted RNA was quantified. To ensure the integrity of the RNA, each sample was run on agarose formaldehyde gel Turano et al. (1997). The first‐strand of the cDNA was made from 1 μg of total RNA of control and treated samples. The cDNA template was synthesized using the reaction mixture containing 20 U/μL Moloney murine leukemia virus reverse transcriptase (MuMLV) enzyme (Fermentas, USA) at 42°C for 50 min and at 70°C for 10 min. The reverse transcription reaction was carried out using 2.5 μM Oligo (dT) 18 primer (Fermentas, USA) and 10 mM dNTPs. Primers for gene expression analysis were designed using online primer designing software (IDT) and cDNA sequences of selected genes were obtained from NCBI.

*Quantitative Real‐Time PCR Analysis*

Real‐time PCR (RT‐PCR) was performed in 96‐well reaction plate (Roche, Germany) containing 20 μL reaction mixture of × 10 reaction buffer, 2 mM dNTPs, 1 mM MgCl_2_, 0.35 μM each of forward and reverse primers, 1 μL Sybr green (×10), 10 μg cDNA template and 5 U Taq polymerase on a thermal cycler (Light cycler 480 II, Roche, Germany). All quantifications were normalized to actin DNA fragment amplified by β‐actin forward and β‐actin reverse primers. The actin gene was used as an internal control for evaluating the efficiency of RT‐PCR for genes. PCR cycling conditions were as follows: denaturation at 95 °C for 3 min, 40 cycles of 95°C (20 s), 66 °C (1 min) and 72 °C (1 min) with 5 min of final extension at 72 °C. The amplified product was resolved on 1.2% agarose gel. The specificity of amplicons was verified by melting curve analysis (60 to 95 °C) after 40 cycles. All reactions were performed in three biological replicates (with three technical replicates of each), using gene‐specific primers and actin primers as an internal control. The data were taken as the expression of the gene of interest in relation to the internal control in the treated sample compared with the untreated control.

**Reference**

1. Beyer Jr, W.F.; Fridovich, I. Assaying for superoxide dismutase activity: some large consequences of minor changes in conditions. *Anal. Biochem.* **1987**, *161*, 559-566.
2. Giannopolitis, C.N.; Ries, S.K. Superoxide dismutases: I. Occurrence in higher plants. *Plant Physiol.* **1977**, *59*, 309-314.
3. Nakano, Y.; Asada, K. Hydrogen peroxide is scavenged by ascorbate-specific peroxidase in spinach chloroplasts. *Plant Cell Physiol.* **1981**, 22, 867-880.
4. Foyer, C.H.; Halliwell, B. The presence of glutathione and glutathione reductase in chloroplasts: a proposed role in ascorbic acid metabolism. *Planta* **1976**, *133*, 21-25.
5. Turano, F.J.; Thakkar, S.S.; Fang, T.; Weisemann, J.M. Characterization and expression of NAD (H)‐dependent glutamate dehydrogenase genes in Arabidopsis. *Plant Physiol.* **1997**, *113*, 1329‐1341.

**Table S1**. Primer pairs used for quantitative RT‐PCR

| S.No. | Gene | Gene ID | Forward primer | | Reverse primer |
| --- | --- | --- | --- | --- | --- |
| 1. | *ACS* | 543237 | GGGTGCATGCGATTGTTTTG | | GTGGTACAACATGGACCTAGAC |
| 2. | *GR* | 123146096 | GCCATGTGTGGACCAGATGC | | GCAGAAGGGTGGATCCCGAC |
| 3. | *PsbA* | 803183 | ATATTGTGGCCGCTCAT | | TCCGTTTAGATTGAAAGCCA |
| 4. | *PsbB* | 803181 | GCCGGAACTATGTGGTAT | | GACCAAGCTTCTGATAAAC |
| Reference gene primer sequences used for quantitative RT-PCR | | | | | |
| 1. | *Actin* | 123048645 | GACTGCCAAGACCAGCTCC | CTTCCTAATATCCACGTCGCAC | |
